# The ric-8b protein (resistance to inhibitors of cholinesterase 8b) is key to preserving contractile function in the adult heart

**DOI:** 10.1016/j.jbc.2024.107470

**Published:** 2024-06-13

**Authors:** Elena Tsisanova, Muriel Nobles, Sonia Sebastian, Keat-Eng Ng, Alison Thomas, Lee Scott Weinstein, Patricia B. Munroe, Andrew Tinker

**Affiliations:** 1 Department of Clinical Pharmacology and Precision Medicine, William Harvey Research Institute, Barts and The London Faculty of Medicine and Dentistry, Queen Mary University of London, London, UK; 2 Building NIHBC 10 - Clinical Center, Bethesda, Maryland, USA

**Keywords:** Ric-8b, stimulatory G-protein, cardiac contraction, calcium channel, myosin light chain 2

## Abstract

Resistance to inhibitors of cholinesterases (ric-8 proteins) are involved in modulating G-protein function, but little is known of their potential physiological importance in the heart. In the present study, we assessed the role of resistance to inhibitors of cholinesterase 8b (Ric-8b) in determining cardiac contractile function. We developed a murine model in which it was possible to conditionally delete *ric-8b* in cardiac tissue in the adult animal after the addition of tamoxifen. Deletion of *ric-8b* led to severely reduced contractility as measured using echocardiography days after administration of tamoxifen. Histological analysis of the ventricular tissue showed highly variable myocyte size, prominent fibrosis, and an increase in cellular apoptosis. RNA sequencing revealed transcriptional remodeling in response to cardiac *ric-8b* deletion involving the extracellular matrix and inflammation. Phosphoproteomic analysis revealed substantial downregulation of phosphopeptides related to myosin light chain 2. At the cellular level, the deletion of *ric-8b* led to loss of activation of the L-type calcium channel through the β-adrenergic pathways. Using fluorescence resonance energy transfer-based assays, we showed ric-8b protein selectively interacts with the stimulatory G-protein, Gαs. We explored if deletion of *Gnas* (the gene encoding Gα_s_) in cardiac tissue using a similar approach in the mouse led to an equivalent phenotype. The conditional deletion of the Gα_s_ gene in the ventricle led to comparable effects on contractile function and cardiac histology. We conclude that ric-8b is essential to preserve cardiac contractile function likely through an interaction with the stimulatory G-protein and downstream phosphorylation of myosin light chain 2.

G protein–coupled receptors, and those whose signaling is mediated by stimulatory G-protein subunit (Gα_s_), play a key role in cardiovascular function such as modulating heart rate, cardiac contractility, and vascular tone. It is apparent that in addition to G protein–coupled receptors and heterotrimeric G-proteins, other proteins can influence the expression of G-proteins and regulate the G-protein cycle. Resistance to inhibitors of cholinesterase proteins were originally identified using a genetic screening in *Caenorhabditis elegans* and reported to function as a novel G-protein regulator (1). These proteins act as chaperones and stabilize the expression of the G-protein alpha (Gα) subunits (2, 3, 4). In addition, the ric-8 proteins, which include two members, ric-8a and ric-8b, may function as a nonreceptor guanine nucleotide exchange factors for Gα subunits (4, 5, 6). Ric-8b has been identified as positively regulating Gα_s_ (7). The ric-8 proteins seem essential for embryonic development, and deletion in the mouse is found to be lethal (8, 9). This has resulted in difficulties in ascertaining their *in-vivo* function.

We have recently shown in the mouse that Ric-8b in the sinoatrial node may be important in the control of heart rate; studies prompted by association of a locus including the gene in a genome wide association study of heart rate (10). This effect was likely mediated by impaired Gα_s_ function and consequent changes in adrenergic signaling in the pacemaker tissues. It is known that adrenergic signaling is critical to normal contractile function and is remodeled in heart failure (11, 12). This prompted us to ask what the consequences for broader heart function might be if ric-8b were deleted in the cardiac ventricles. In this study, we have developed a murine model in which it is possible to conditionally delete ric-8b throughout the heart using the cre∖loxP approach to address this question. We also compared these mice to an equivalent line with deletion of Gnas which encodes Gαs.

## Results

### Characterization of Ric-8b study mice

**Figure 1:**
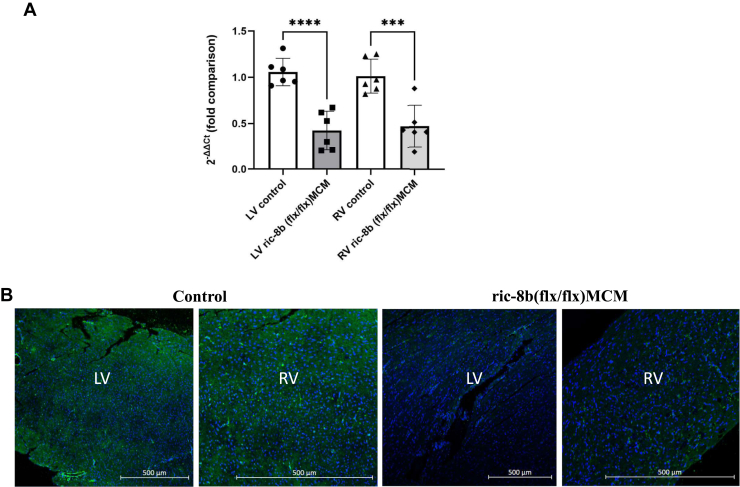
**Deletion of ric-8b in cardiac tissue.***A*, quantitative real time PCR in left (LV) and right (RV) ventricle. N = 6 mice for both groups (control group: three WT and 3 cre+). ∗∗∗*p* < 0.001, ∗∗∗∗*p* < 0.0001 one-way ANOVA, Turkey's multiple comparison test. *B*, expression of ric-8b in cardiac tissue using antibody staining. Images are representative of similar staining performed in three mice in each group (control: two WT and one cre+).

We crossed mice to generate *ric-8b (flx/flx)* combined with the cardiac muscle specific tamoxifen inducible cre referred to as “*ric-8b (flx/flx) MCM”* below and in the Figures. Mice were then studied at 10 to 12 weeks of age. In pilot experiments, 5 days of tamoxifen injection in *ric-8b (flx/flx)* mercremer (*MCM*) led to significant mortality within the next couple of days. Thus, we adopted a 3-day injection protocol with a 48 h study after the last injection of tamoxifen, and this enabled us to examine the acute effects of cardiac deletion of ric-8b. Control animals are littermates with a mix of either a MCM positive or normal genotype. We performed quantitative real-time RT-PCR on RNA extracted from left and right ventricles. *Ric-8b* RNA expression was significantly decreased in both left and right ventricles after tamoxifen treatment compared to control mice (Fig. 1*A*). We also used immunofluorescence in cardiac tissue sections and ric-8b staining was reduced after tamoxifen treatment (Fig. 1*B*).

### Echocardiography of ric-8b (flx/flx)MCM hearts

**Figure 2:**
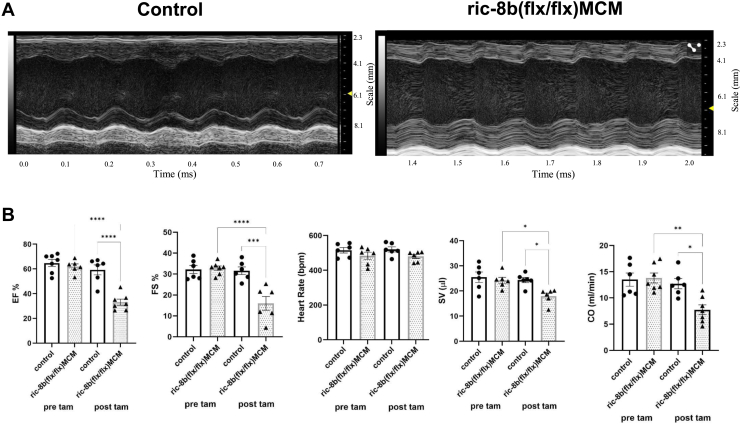
**Cardiac echography after deletion of ric-8b.***A*, representative echocardiography image from a control and a *ric-8b (flx/flx)MCM* mice. *B*, ejection fraction, cardiac output, fractional shortening, heart rate, and stroke volume before and after tamoxifen treatment in control (two WT and four cre+) and, *ric-8b (flx/flx)MCM*. N = 6 mice for both groups. ∗*p* < 0.05, ∗∗*p* < 0.01, ∗∗∗*p* < 0.001, ∗∗∗∗*p* < 0.0001. one -way ANOVA, Tukey's multiple comparison test. MCM, mercremer.

The echocardiographic assessment of the hearts of *ric-8b (flx/flx) MCM* mice revealed profound changes 2 days after tamoxifen treatment (Fig. 2 and Table 1). Tamoxifen had no significant effects in the control animals (Fig. 2 and Table 1). However, in the *ric-8b (flx/flx)MCM* animals ejection fraction, fractional shortening and cardiac output all fell after treatment with tamoxifen (Fig. 2 and Table 1). The fall in cardiac output was due to a fall in stroke volume and was accompanied by an increase in lumen dimensions (Table 1).

**Table 1:** Echocardiographic parameters in the ric-8b (flx/flx)MCM mice (n = 6) and control (n = 6) before and after tamoxifen

ECHO parameter	Control		Ric-8b flx/flxMCM	
	Pre-TAM	Post TAM	Pre-TAM	Post TAM
Ejection fraction, %	66.83 ± 2.34	59.25 ± 4.05	61.96 ± 2.11	33.64 ± 2.9∗∗
Cardiac output, ml/min	13.50 ± 1.26	12.73 ± 0.97	13.80 ± 0.95	7.75 ± 0.94∗∗
Heart rate, bpm	515.6 ± 15.62	520.5 ± 14.56	483.7 ± 20.47	480.4 ± 11.90
Fractional shortening, %	32.20 ± 1.94	31.62 ± 1.84	32.78 ± 1.10	16.01 ± 3.26∗
Stroke volume, μl	25.50 ± 2.09	24.36 ± 1.05	24.23 ± 1.22	17.99 ± 1.18∗
LV mass, mg	85.81 ± 6.93	98.63 ± 6.26	113.8 ± 10.4	169.4 ± 24.69
IVS;s	1.28 ± 0.06	1.13 ± 0.15	1.274 ± 0.08	1.07 ± 0.20
IVS;d	1.06 ± 0.21	0.83 ± 0.13	0.95 ± 0.22	1.2 ± 0.28
LVID;s	2.37 ± 0.17	2.49 ± 0.37	2.24 ± 0.14	3.2 ± 0.23∗∗
LVID;d	3.34 ± 0.12	3.38 ± 0.33	3.32 ± 0.13	3.50 ± 0.21
LVPW;s	1.25 ± 0.16	1.07 ± 0.16	1.25 ± 0.06	0.98 ± 0.08
LVPW;d	0.94 ± 0.12	0.84 ± 0.14	0.94 ± 0.05	0.95 ± 0.08

LV mass, left ventricular mass; IVS;s, interventricular septum in systole; IVS;d, interventricular septum in diastole; LVID;s, left ventricular internal diameter in systole; LVID;d, left ventricular internal diameter in diastole; LVPW;d, left ventricular posterior wall in systole; LVPW;d, left ventricular posterior wall in diastole. ∗*P* < 0.05, ∗∗*P* < 0.01 paired t test.

### Cardiac morphology and histology of ric-8b (flx/flx)MCM mouse

**Figure 3:**
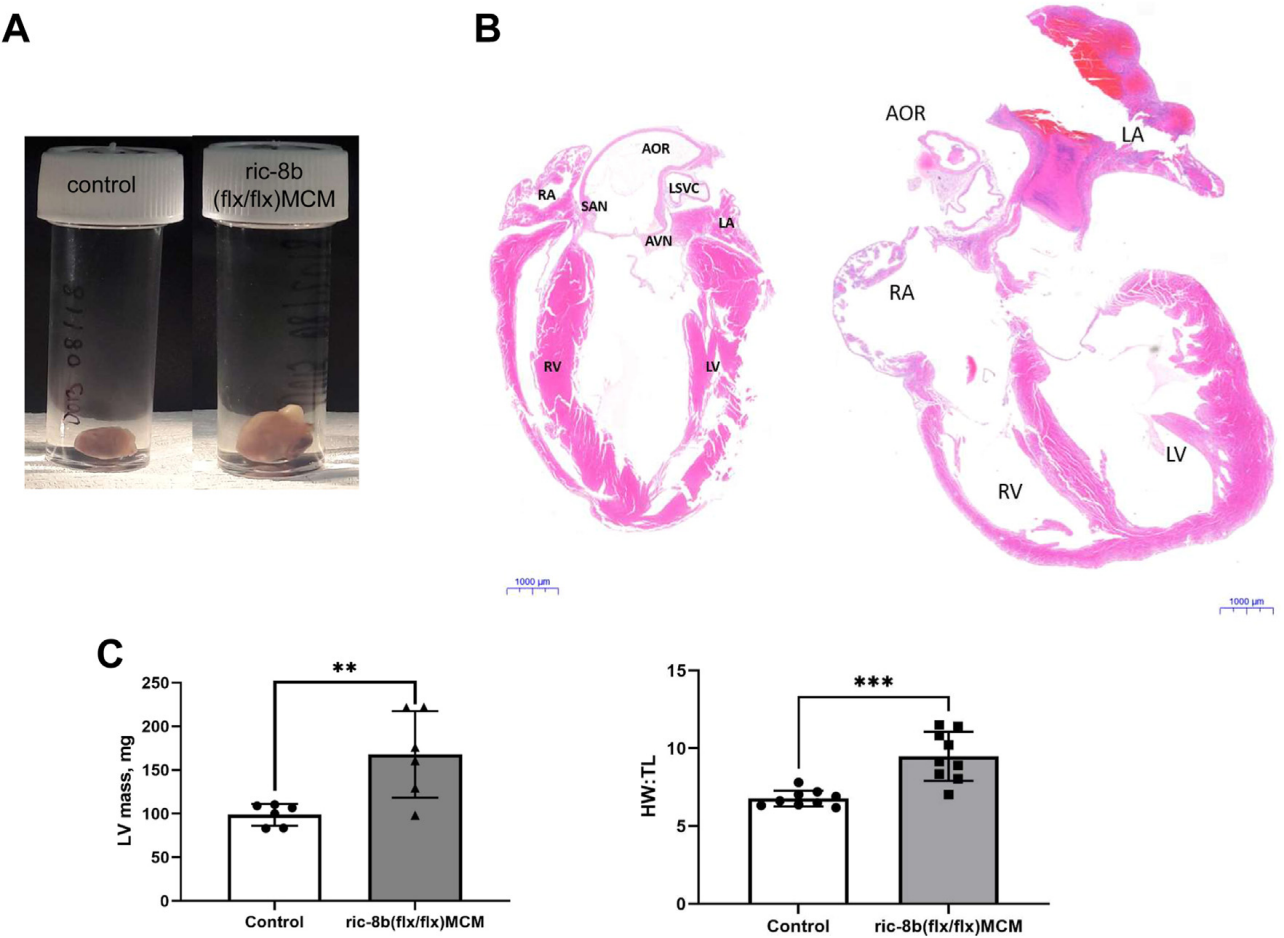
**Cardiac hypertrophy after deletion of ric-8b.***A*, enlarged heart pictured (*left*) after deletion of ric-8b and, cross section of the cardiac tissue showing enlarged ventricles (especially LV). Images representative of similar observations in three mice in each group. *B*, H&E-stained heart sections from control (2 WT and 1 cre+) and, ric*-8b (flx/flx)MCM* mouse (the scale bar represents 1000 μm). Images representative of similar staining performed in three mice in each group. *C*, increased LV mass of ric*-8b (flx/flx)MCM* mice performed during echocardiography recording (*left*), n = 6 mice both groups (control group (2 WT and 4 cre+). ∗∗*p* < 0.01, unpaired *t* test; Comparison of heart weight *versus* tibial length of control (five WT and four cre+)and ric-8b (flx/flx)MCM mice (*right*), n = 9 mice both groups, ∗∗*p* < 0.01, unpaired *t* test. LV, left ventricular; MCM, mercremer.

**Figure 4:**
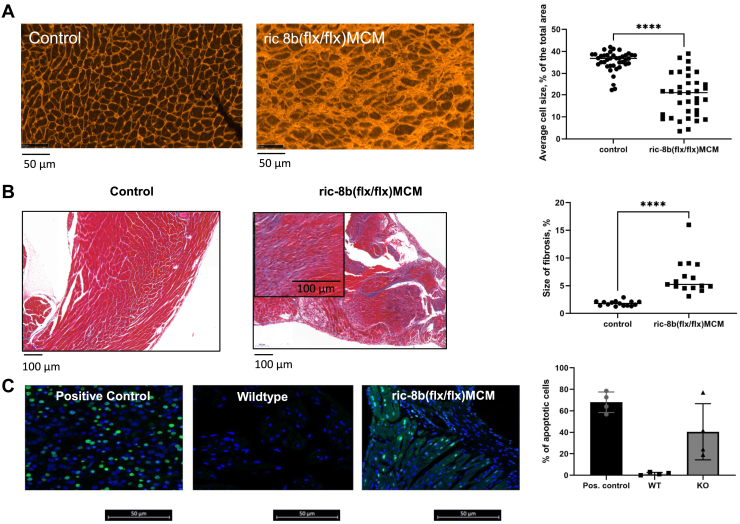
**Cardiac histology after deletion of ric-8b.***A*, paraffin-embedded whole heart tissues stained with wheat germ agglutinin, showing disorganized tissue after deletion of ric-8b. Images are representative of similar staining performed in three mice in each group. Quantification performed in sections from three mice in each group (Control: two WT and one cre+). *B*, longitudinal sections of whole hearts stained with trichrome staining showing an increase in fibrotic tissue after deletion of ric-8b. *Blue*, fibrillar collagen; *red*, myocardium. Images are representative of similar staining performed in three mice in each group. Quantification performed in sections from three mice in each group (Control: two WT and one cre+). *C*, cardiac apoptosis after deletion of ric-8b. TUNEL staining showing an increase in apoptotic cells after deletion of ric-8b. Images are representative of similar staining performed in three mice in each group. Quantification performed in sections from three mice in each group (Control: two WT and one cre+). ∗∗∗*p* < 0.001, ∗∗∗∗*p* < 0.0001, one-way ANOVA, Tukey's multiple comparison test.

When the heart was examined 2 days after the end of treatment with tamoxifen, there was evidence of cardiac dilatation on gross histological examination (Fig. 3, *A* and *B*). The heart was enlarged with an increase in left ventricular (LV) mass as calculated from echocardiography (Fig. 3*C* left panel and Table 1) and an increase in heart weight to tibial length (Fig. 3*C* right panel). Cardiac histological examination showed increased variability of myocyte size after wheat germ agglutinin (WGA) staining in *ric-8b (flx/flx)MCM* mice after treatment with tamoxifen compared to control mice and increased extracellular matrix (Fig. 4*A*). There was also increased fibrosis in the ventricles of *ric-8b (flx/flx)MCM* mice after treatment with tamoxifen (Fig. 4*B*). Finally, the use of the TUNEL assay revealed a marked increase in apoptotic cells with fragmented DNA throughout the cardiac tissue of *ric-8b (flx/flx)MCM* mice after tamoxifen treatment (Fig. 4*C*). Apoptotic cells were not present in the hearts of control mice (Fig. 4*C*).

### RNA sequencing

**Figure 5:**
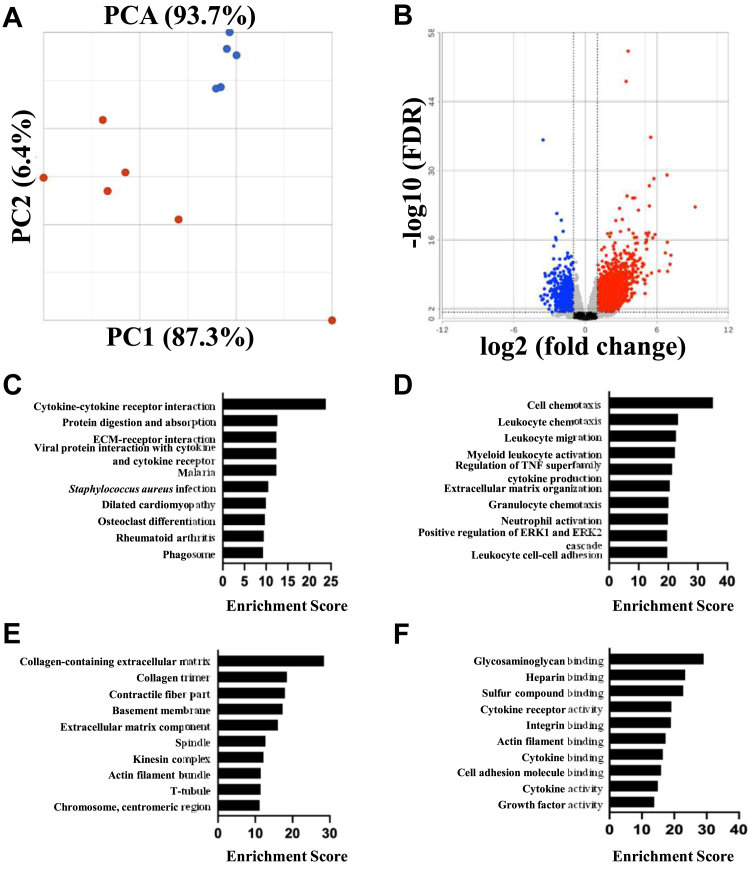
**RNA sequencing analysis.***A*, principal component analysis of control (CON, n = 5, *blue dots*, four WT and one cre+) and r*ic-8b (flx/flx)MCM* (KO, n = 6, *red dots*) mice. *B*, volcano plot of differential expression analysis. Log2fold change (Log2FC) is plotted on the *x*-axis and –log10 (FDR) is plotted on the *y*-axis. Significantly downregulated genes are indicated in *blue* and upregulated genes in *red*, log2FC < −1 or >1, respectively. Pathways enriched in *ric-8b (flx/flx)MCM* mice. *C*, top 10 KEGG and the top 10 Gene ontology pathways, (*D*) biological process (BP), (*E*) cellular component (CC) and (*F*) molecular function (MF). FC, fold change; FDR, false discovery rate; KEGG, Kyoto Encyclopedia of Genes and Genomes.

We extracted RNA from the ventricles of *ric-8b (flx/flx)MCM* (n = 6) and control mice (n = 5) 2 days after the end of tamoxifen treatment and performed RNA sequencing (Fig. 5 and supplementary Data). A total of 2840 genes were identified to show significant differential expression (false discovery rate, (FDR) < 0.05 and a log fold change (logFC) of ≤ −1 and ≥ 1). Of these, 1869 genes were found to be upregulated (logFC ≥ 1) in *ric-8b* (*flx*/*flx*)*MCM* mice, whereas 971 genes were found to be downregulated (logFC ≤ −1) including *ric-8b* (FDR 4.30 × 10^−22^) (Fig. 5*B* and Tables S1 and S2). Other genes found to be downregulated in *Ric-8b* (*flx*/*flx*)*MCM* mice include the L-type Ca^+^ channel, *Cacna1c* (FDR 1.16 × 10^−7^), ryanodine receptors, *Ryr2* and *Ryr3* (FDR 6.31 × 10^−10^ and 3.67 × 10^−6^, respectively) and adenylate cyclases, *Adcy1* and *Adcy9* (FDR 2.23 × 10^−3^ and 3.99 × 10^−3^, respectively) (Table S2). Genes that were upregulated in r*ic-8b* (*flx*/*flx*)*MCM* mice include those encoding collagen including collagen type III alpha I (*Col3a1*, FDR 6.94 × 10^–6^) as well as fibrosis markers, such as connective tissue growth factor (*Ccn2*, FDR 5.04 × 10^–14^) and transforming growth factor beta1 (*Tgfb1*, FDR 3.23 × 10^–3^) (Table S1). We confirmed the increased expression of a number of the fibrosis genes using quantitative PCR (qPCR) (Supporting Figure). Gene set enrichment analysis using Kyoto Encyclopedia of Genes and Genomes (KEGG) and Gene Ontology databases indicate that there is an enrichment in genes accounting for extracellular matrix organization, inflammation, and the cell cycle (Fig. 5, *C*–*F*, Tables S3, and S4). Further analysis using only upregulated genes as input show enrichment within the cell cycle pathway, whereas downregulated genes are enriched in metabolic and signaling pathways (Tables S5 and S6). In summary, there are a number of transcriptional changes in the heart in response to the deletion of ric-8b in the heart including changes in genes in the cell cycle, metabolism, and cell signaling pathways.

### Phosphoproteomics

**Figure 6:**
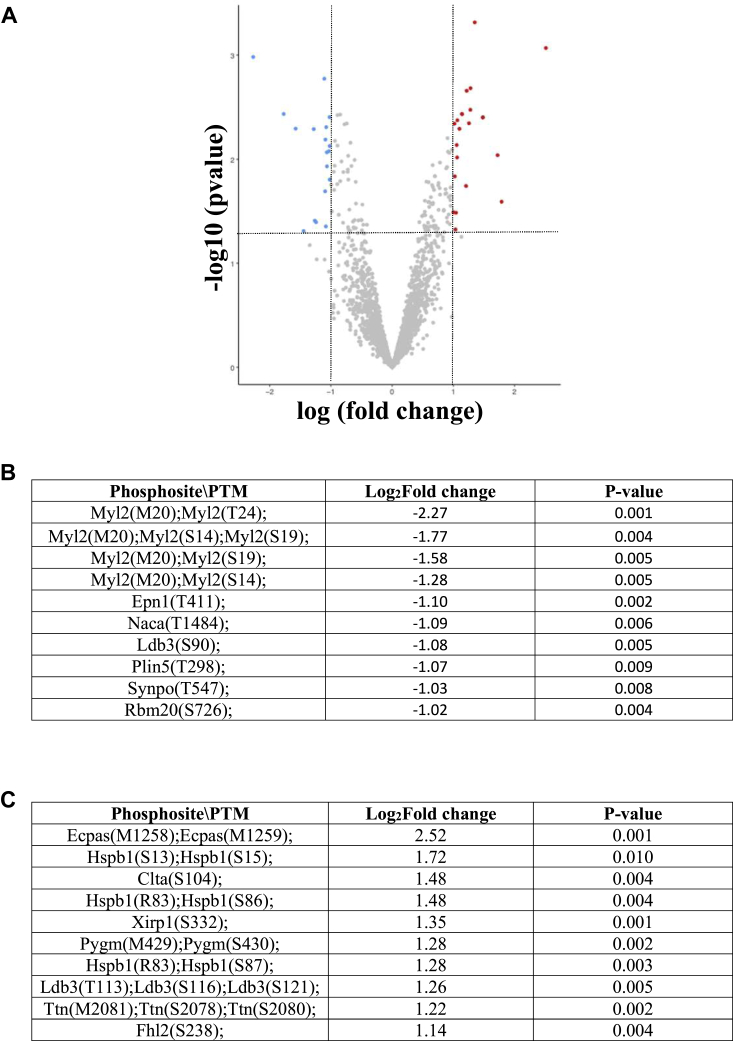
**Phosphoproteomic analysis.** N = 4 mice in both groups (two WT and two cre+). *A*, volcano plot of phosphoprotein analysis. Log fold change (LogFC) is plotted on the *x*-axis and –log10 (*p*-value) is plotted on the *y*-axis. Significantly downregulated genes are indicated in *blue* and upregulated genes in *red*, logFC < −1 or >1, respectively. *B*, top 10 downregulated phosphopeptides. *C*, top 10 upregulated phosphopeptides. FC, fold change; MCM, mercremer.

Given the role of ric-8b in G-protein signaling, we analyzed the potential differential phosphorylation of downstream proteins in an unbiased fashion using phosphoproteomic mass spectrometry (MS) (see Experimental procedures) from the ventricles of *ric-8b (flx/flx)MCM* and control mice (n = 4 both groups) 2 days after the end of tamoxifen treatment. These were different groups of mice from those used for the RNA-seq analysis. Nineteen phosphopeptides were significantly downregulated while twenty were significantly upregulated, and the top 10 phosphopeptides in both categories are shown in Figure 6. It is clear that myosin light chain 2 is a major target that has reduced phosphorylation. Using kinase substrate enrichment analysis, *i.e.* the potential change in kinase activity identified from the changes in substrate phosphorylation, we identified protein kinase C alpha and delta (prkca and prkcd) and mitogen-activated protein kinase 1 (mapk1) and mitogen-activated protein kinase kinase kinase 7 (map3k7) as being significantly (z score>2) upregulated between KO mice and WT. We performed pathway analysis using KEGG and found seven pathways that were significantly downregulated and another seven that were significantly upregulated (Tables S7 and S8). In summary, the results point to the key role of ric-8b in cardiac morphogenesis, sarcomere organization and cardiac contraction likely through the phosphorylation of myosin light chain 2.

### Localization and consequences for signaling of ric-8B in ventricular cardiomyocytes

**Figure 7:**
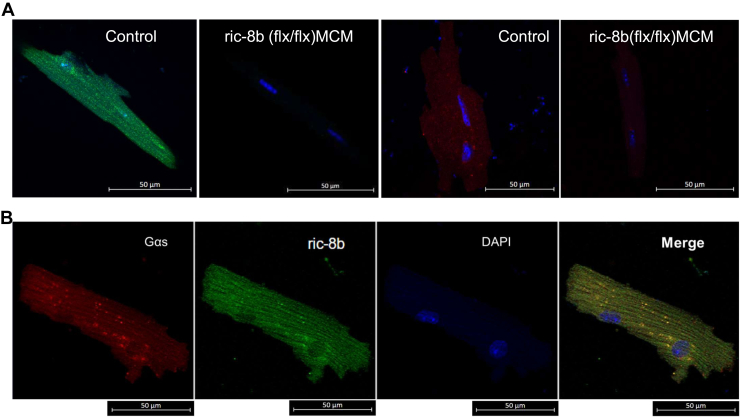
**Expression of Ric-8B and Gα**_**s**_**in isolated ventricular cardiomyocytes using antibody staining.***A*, *top panel*: expression of ric-8b (1:100 Ric-8b rabbit 1° antibody (ab170006, Abcam) and 1:500 2° anti-rabbit Alexa Fluor 488 antibody) and Gα_s_ (1:10 Gα_s_ sc-55545, Santa Cruz Biotechnology) and 1:500 2° anti-mouse Alexa Fluor 594 antibody) in fixed ventricular myocytes from control and *ric-8b (flx/flx)MCM* mice. *B*, expression of Gα_s_, ric-8B in fixed ventricular myocytes from control mouse (1:100 ric-8b rabbit 1° antibody (ab170006, Abcam) and 1:500 2° anti-rabbit Alexa Fluor 488 antibody) and Gα_s_ ((1:10 Gα_s_ sc-55545, Santa Cruz Biotechnology) and 1:500 2° anti-mouse Alexa Fluor 594 antibody). Images are representative of similar staining performed in three mice in each group (control: two WT and one cre+). MCM, mercremer

Given the previously described interaction of ric-8b with Gαs, we examined the distribution of these two proteins in the cardiac cell. Ventricular cardiomyocytes were isolated from control and *ric-8b (flx/flx)MCM* mice. When myocytes were stained with a Ric-8b antibody, Ric-8b was present throughout the cells from WT mice, and practically absent in the KO mice (Fig. 7*A*). It was only possible to stain the cardiomyocytes from control mice with Gαs antibody as we were unable to detect staining of Gαs in *ric-8b (flx/flx)MCM* mice (Fig. 7*A*). In control mice, Gαs colocalized with ric-8b, (Fig. 7*B*, overlap coefficient 0.85 ± 0.03, n = 3).

**Figure 8:**
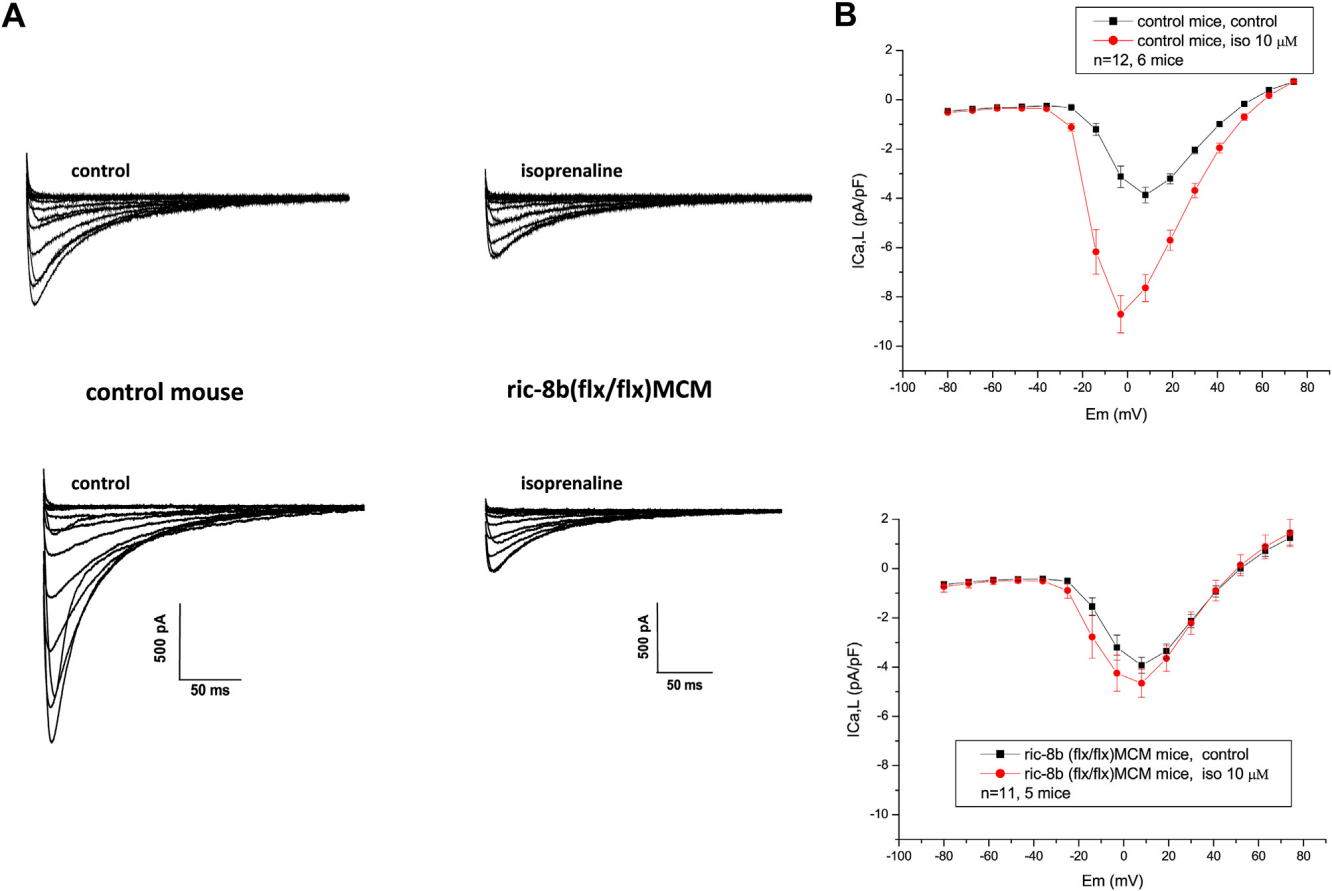
**Modulation of ICa,L by ric-8B.** Electrophysiology of cardiac calcium current (ICa,L) measured in isolated ventricular myocytes using the whole-cell configuration of the patch-clamp method. *A*, representative traces of ICa,L in control conditions, comparison between control and *ric-8b* (*flx/flx)MCM* mice. *B*, currents measured in control and after 10 μM isoprenaline in control cardiomyocytes (*upper panel*, n = 12 cells from six mice (three WT and three cre+)) and currents measured in control and after 10 μM isoprenaline in *ric-8b (flx/flx) MCM* cardiomyocytes (*lower panel*, n = 11 cells from five mice). Basal currents measured at +8 mV were not significantly different between the two groups. However, currents evoked by isoprenaline were significantly larger compared to control currents in the control mice (∗∗∗∗*p* < 0.0001 paired *t* test) but were not increased in *ric-8b (flx/flx)MCM* mice. MCM, mercremer.

We then investigated Gαs signaling in ventricular cardiomyocytes from control and *ric-8b (flx/flx)MCM* mice. Specifically, we focused on the modulation of L-type calcium currents (ICa,L) by beta adrenergic receptor stimulation. We compared the ICa,L currents in ventricular myocytes from control mice and r*ic-8b (flx/flx)MCM* mice. The basal calcium currents, measured at −3 mV, were similar in cells expressing Ric-8b (−3.12 ± 0.43pA/pF, n = 12) or in cells lacking ric-8b (−3.92 ± 0.32, n = 11, NS, Fig. 8). However, when isoprenaline was applied to the cells, there was a significant increase in the ICa,L currents in the cardiomyocytes from control mice (−8.7 ± 0.75 pA/pF, n = 12, *p* < 0.0001), but no significant increase in cardiomyocytes lacking ric-8b, −4.65 ± 0.56 pA/pF, n = 11, Fig. 8).

### Interaction of ric-8B protein with Gα_s_ visualized by FRET

**Figure 9:**
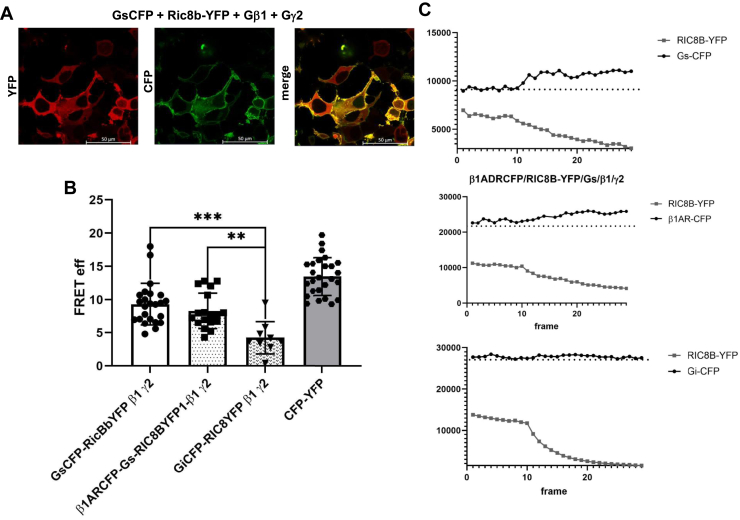
**Interaction of ric-8B and Gαs: FRET using acceptor photobleaching.** Interaction of ric-8B and Gα_s_ in HEK cells expressing Gα_s_-CFP, β1-adrenergic receptor (B1-AR), Gβ1, Gγ2 and, ric-8B-YFP. *A*, representative FRET image (FRET between Gα_s_-CFP and ric-8B-YFP). *B*, summary of the data (n = 15). One-way ANOVA, Tukey’s multiple comparison test,∗∗ *p* < 0.01, ∗∗∗ *p* < 0.001; (*C*) from *top to bottom*: Representative FRET photobleaching traces between Gα_s_-CFP and ric-8B-YFP; Gα_i_-CFP and ric-8B-YFP; β1-AR and ric-8B-YFP. YFP, yellow fluorescent protein; CFP, cyan fluorescent protein.

To study the potential interaction between ric-8B and Gαs, we used fluorescently tagged constructs, specifically fusions with the cyan fluorescent proteins (CFPs) and yellow fluorescent proteins (YFPs), of ric-8b, G-protein alpha subunits and the β1 adrenergic receptor. These complementary DNAs (cDNAs) were transfected into HEK293 cells and FRET efficiency was measured using acceptor photobleaching. When Ric-8b-YFP, Gαs-CFP, Gβ1, and Gγ2 (G-protein beta and gamma subunits) were transfected into HEK293 cells they were largely colocalized (Fig. 9*A*) as with the native proteins in ventricular myocytes. It should be noted in some cytoplasmic regions that there was ric-8b-YFP signal that was not accompanied by significant Gαs-CFP fluorescence. Photobleaching of ric-8b-YFP led to an increase in Gαs-CFP fluorescence indicative of FRET (Fig. 9*B*). The efficiency was equivalent to that of a CFP-YFP dimer that we have used as a positive control (Fig. 9*C*). To examine the specificity of the effect, we transfected ric-8b-YFP, Gα_i2_-CFP, Gβ1, and Gγ2 into HEK293 cells. In these experimental conditions, FRET was attenuated between ric-8b-YFP and Gα_i2_-CFP compared to signal from the ric-8b-YFP and Gαs-CFP pairing, (Fig. 9*C*). These observations can be interpreted as showing a specific interaction between Ric-8b and Gαs proteins over those with inhibitory G-proteins specifically Gα_i2_. In addition, we transfected ric-8b-YFP together with a tagged version of the beta-1 adrenergic receptor (β1AR-CFP) into HEK293 cells. Under these conditions, there was also a significant FRET signal between the two proteins (Fig. 9*B*), and this is discussed below.

**Figure 10:**
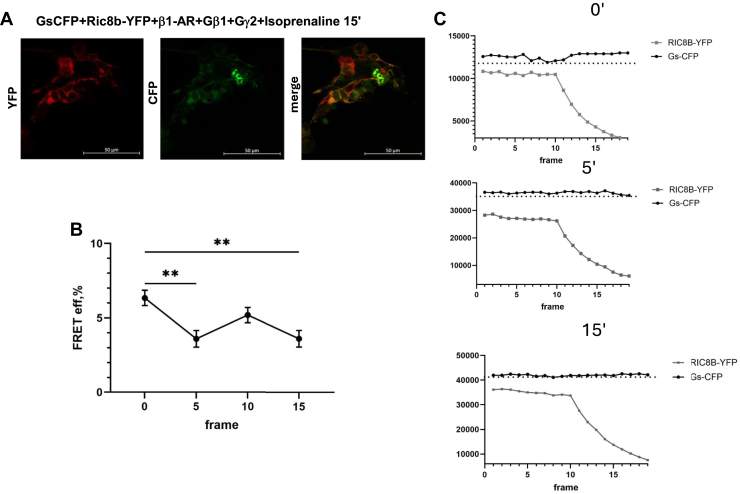
**Interaction of ric-8B and Gαs in the presence of β1-adrenergic receptor agonist.***A*, representative image of FRET 15 min after the addition of 10 μM isoprenaline. *B*, FRET efficiency time-course (at 0, 5, 10, and 15 min after the addition of 10 μM isoprenaline) (n = 6), FRET between Gα_s_-CFP and ric-8B-YFP in the presence of β1-AR from top to 5 min after the addition of the agonist. One-way ANOVA, Tukey's multiple comparison test, ∗*p* < 0.05, ∗∗*p* < 0.01. *C*, from *top to bottom*: representative FRET photobleaching traces between Gα_s_-CFP and ric-8B-YFP at 0, 5, and 15 after addition of 10 μM isoprenaline. CFP, cyan fluorescent protein;YFP, yellow fluorescent protein.

We explored the role that agonist activation might have by expressing beta 1-adrenergic receptor and adding isoprenaline. We transfected ric-8b-YFP together with a with Gαs-CFP and untagged beta1-adrenergic receptor (β1-AR) into HEK293 cells. Under these conditions, we could measure a FRET signal between the two proteins before addition of 10 μM isoprenaline as above. FRET values were then also measured after 5, 10, and 15 min (Fig. 10). FRET efficiency fell in the presence of agonist and persisted for 15 min after addition (Fig. 10).

### Echocardiography of Gα_s_ (flx/flx)MCM hearts

**Figure 11:**
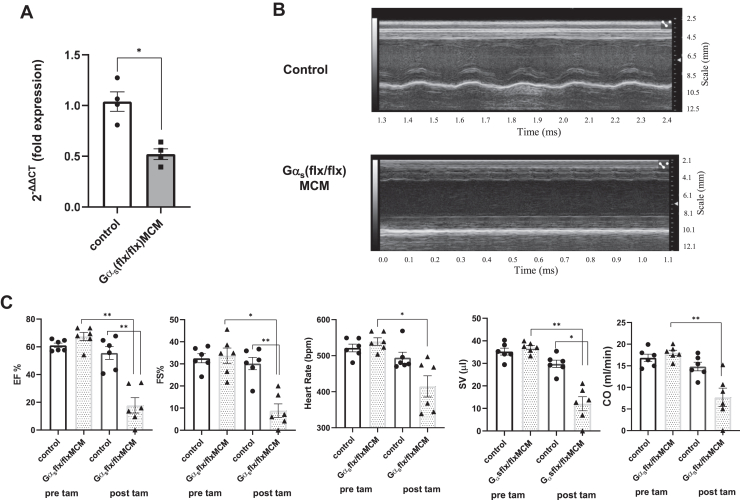
**Cardiac echocardiography after the deletion of Gαs.***A*, qPCR in cardiac tissue after tamoxifen treatment showing decreased expression of Gαs. (n = 4 control (two WT and two cre+), n = 4 *Gα*_*s*_*(flx/flx) MCM* using Mann Whitney non-parametric *t* test. (∗*p* < 0.05). *B*, representative echocardiography image of heart from a *Gα*_*s*_*(flx/flx) MCM* and a control mouse. *C*, ejection fraction, fractional shortening, heart rate, cardiac output, and stroke volume before and after tamoxifen treatment (n = 6 control (four WT and two cre+), n = 6 *Gα*_*s*_*(flx/flx) MCM*) using one-way ANOVA with Sidak’s multiple comparison test. ∗*p* < 0.05 and ∗∗*p* < 0.01. MCM, mercremer; qPCR, quantitative PCR.

Given the interaction between ric-8b and Gαs, we generated another murine model in which it was possible to conditionally delete the stimulatory G-protein in myocytes of the heart after the addition of tamoxifen. The tamoxifen administration protocol was identical to the *ric-8b (flx/flx)MCM* mice, and in addition, we studied the mice 2 days after the final injection. We used qPCR to assess the expression of Gαs after the addition of tamoxifen, and the expression was reduced compared to control mice (Fig. 11*A*).

Cardiac echocardiography of the hearts of *Gα*_*s*_ *(flx/flx)MCM* mice revealed decreases in ejection fraction, fractional shortening, heart rate, stroke volume, and cardiac output after tamoxifen treatment (Fig. 11 and Table 2). Tamoxifen had no significant effects in the control mice (Table 2). There were no significant changes in wall or chamber dimensions (Table 2). It should be noted that there was no hypertrophy of the posterior wall of the ventricle in *Gα*_*s*_ *(flx/flx)MCM* and resulting in poorer chamber definition.

**Table 2:** Echocardiographic parameters in the *Gα*_*s*_*(flx/flx) MCM* mice n = 6 and control n = 6 before and after tamoxifen

ECHO parameters	Control (n = 6)		Gαsflx/flxMCM (n = 6)	
	Pre-TAM	Post TAM	Pre-TAM	Post TAM
Ejection fraction, %	61.0 ± 1.62	55.5 ± 4.58	67.4 ± 2.9	17.8 ± 5.54∗∗
Cardiac output, ml/min	16.8 ± 0.84	14.8 ± 0.99	17.9 ± 0.63	7.7 ± 2.12∗∗
Heart rate, bpm	521 ± 10.6	494 ± 15.2	539 ± 10.3	414 ± 29.4∗
Fractional shortening, %	32.5 ± 2.06	30.14 ± 2.80	33.6 ± 3.48	8.85 ± 3.08∗
Stroke volume, μl	35.2 ± 1.52	29.7 ± 1.65	37.0 ± 0.94	12.0 ± 3.11∗∗
LV mass, mg	101.2 ± 7.8	125.7 ± 10.3	109.8 ± 5.2	130.2 ± 24.5
IVS; s mm	1.25 ± 0.18	1.20 ± 0.13	1.15 ± 0.24	1.27 ± 0.16
IVS; d mm	1.20 ± 0.14	1.14 ± 0.12	1.14 ± 0.14	1.07 ± 0.24
LVID; s mm	3.18 ± 0.15	3.20 ± 0.16	3.36 ± 0.12	3.39 ± 0.14
LVID; d mm	3.53 ± 0.18	3.73 ± 0.20	3.52 ± 0.14	3.35 ± 0.63
LVPW; s mm	1.62 ± 0.13	1.76 ± 0.15	1.60 ± 0.15	1.29 ± 0.62
LVPW; d mm	1.63 ± 0.15	1.54 ± 0.08	1.29 ± 0.17	0.95 ± 0.50

EF%, CO, HR, FS%, and SV all found to be significantly lower in *Gα*_*s*_*(flx/flx) MCM* mice compared to control after tamoxifen, while there was no change in wall dimension measurements in *Gα*_*s*_*(flx/flx) MCM* mice and control mice before and after tamoxifen using paired Student’s *t* test (∗*p* < 0.05, ∗∗*p* < 0.01).

### Cardiac morphology and histology of Gα_s_ (flx/flx)MCM mouse

**Figure 12:**
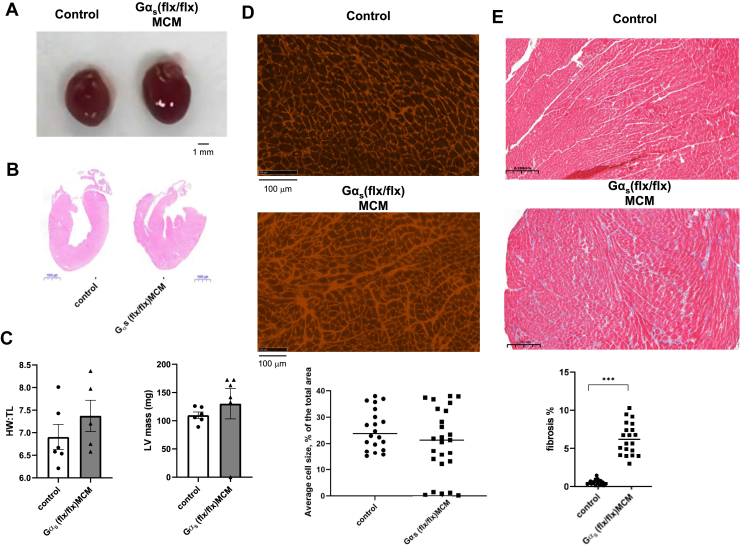
**Cardiac histology after deletion of Gαs.***A*, heart pictured after deletion of Gαs. *B*, H&E stained heart sections from *Gα*_*s*_*(flx/flx) MCM* and control mice. *C*, no increase of cardiac mass (LVmass) (n = 6 control (4 wildtype and 2 cre+), n = 6 *Gα*_*s*_*(flx/flx) MCM* and no change in HW:TL after tamoxifen treatment in *Gα*_*s*_*(flx/flx) MCM* mice (n = 6 control (four WT and two cre+), n = 5 *Gα*_*s*_*(flx/flx) MCM)*. Not significant using Mann Whitney nonparametric *t* test. *D*, wheat germ agglutinin staining of ventricular sections showing variable cell size in *Gα*_*s*_*(flx/flx) MCM* than in control mice. Images are representative of similar staining performed in three mice in each group. Quantification performed in sections from three mice in each group (for the control: two WT and 1 cre+). *E*, paraffin-embedded whole hearts stained with trichrome showing an increase in fibrotic tissue after deletion of *Gnas*. Images are representative of similar staining performed in three mice in each group. Quantification performed in sections from three mice in each group (for the control: two WT and one cre+). MCM, mercremer.

On gross inspection of the hearts, there was no pronounced cardiac dilatation (Fig. 12*A*). The ratio of heart weight to tibial length was similar in control and *Gα*_*s*_ *(flx/flx)MCM* mice while there was a nonsignificant trend to increased cardiac mass as measured using echocardiography (Fig. 12*B*). Cardiac histological examination using WGA staining (Fig. 12*C*) showed highly variable myocyte size. Staining also revealed significantly increased fibrosis in *Gα*_*s*_ *(flx/flx)MCM* hearts after tamoxifen compared to control (Fig. 12*D*). There are both similarities and differences in the phenotypes in the two lines of mice and this is discussed further below.

## Discussion

We have investigated the physiological role of ric-8b in cardiac function, and we show a novel role for ric-8b in preserving contractile function in the adult murine heart.

### Mechanism of cardiac dysfunction

Echocardiography revealed a pronounced decline in contractile function as evidenced by a fall in fractional shortening, ejection fraction, and cardiac output after deletion of ric-8b in cardiomyocytes. This was accompanied by some chamber dilatation both with echocardiography and inspection of the heart after death. This transpired acutely within 48 h of the last tamoxifen dose. If mice were studied beyond this or with a more prolonged tamoxifen administration protocol then significant mortality occurred. This was not formally quantified with survival analysis because of animal welfare considerations. Further histological analysis showed an increase in extracellular matrix and fibrosis and increased apoptotic cell death. This was accompanied by a lack of cellular hypertrophy but an increase in cardiac mass. Synthesizing these observations, it is plausible that increased cell death leads to replacement with extracellular matrix without pronounced cellular hypertrophy, and this leads to an increase in cardiac mass. The acute myocyte loss results in contractile dysfunction.

### Ric-8b and stimulatory G-protein signaling

Ric-8b can clearly influence stimulatory G-protein signaling likely as an important chaperone for Gαs and also perhaps as a guanine nucleotide exchange factor independent of GPCR signaling (13, 14). Our data strongly support this paradigm. In native and heterologous systems, we observed colocalization of ric-8b and Gαs. In our murine model, the loss of Ric-8b led to difficulty in detecting of Gαs in cardiomyocytes. Furthermore, in living cells using heterologously expressed fusions of the proteins with fluorescent proteins, we were able to detect fluorescence resonance energy transfer between ric-8b and Gα_s_ indicative of the formation of a protein complex. This complex was not present when a member of the inhibitory G-protein family was used. Furthermore, if the beta1 adrenergic receptor was cotransfected, the complex destabilized between ric-8b and Gα_s_ after the addition of the beta receptor agonist, isoprenaline. One interpretation of these results is that some ric-8b is displaced from the stimulatory G-protein during active signaling perhaps by interacting with known effectors such as adenylate cyclase. Finally, there also seemed to be FRET between a tagged version of the beta1 receptor and ric-8b in the absence of agonist. We have previously shown a degree of preassembly of receptor and heterotrimeric G-protein in living cells, and the interaction between Ric-8b and beta1 adrenergic receptor may reflect this phenomenon (15).

We also showed loss of beta-adrenergic modulation of L-type calcium channels in cardiomyocytes lacking ric-8b. This regulation is a canonical siganaling pathway mediated by the stimulatory G-protein. Thus, a second consideration in the development of cardiac contractile dysfunction may be the loss of Gα_s_ mediated signaling. This molecular event is critical for the potentiation of contractility *via* sympathetic stimulation. Accordingly, another factor may be the lack of compensatory increase in adrenergically mediated inotropy as LV function declines (16). This is discussed in more detail below.

### Comparison of phenotypes between Ric-8b and Gα_s_ KO mice

A component of the phenotype is likely accounted for by the actions of ric-8b on Gα_s_ signaling, but it remains possible that ric-8b has functions independent of its actions on heterotrimeric G-protein signaling. Thus, we developed an additional murine model in which we replicated the strategy for gene deletion in the adult animal but this time deleting Gα_s_ and studied contractile function and heart histology 2 days after the last tamoxifen injection. We observed some similarities in phenotype in particular a pronounced reduction in fractional shortening, ejection fraction, and cardiac output with increased extracellular matrix and fibrosis in Gα_s_ KO mice. The decline in contractile function was more severe in the Gα_s_ KO mice (Gα_s_-ejection fraction: 67% prior to tamoxifen, 18% after tamoxifen, a 73% reduction, Ric-8b–ejection fraction 62% prior to tamoxifen, 34% after tamoxifen, a 45% reduction). In contrast, in the Gα_s_ KO mice there were no increases in cardiac mass and chamber dimensions. In addition, the heart rate is reduced with Gα_s_ deletion, but this did not occur with ric-8b deletion. We know from our previous studies that haploinsuffciency of ric-8b in the sinoatrial node can lead to changes in heart rate (10). These dissimilarities may be due to differences in efficiency of this cre for different “floxed” alleles in the conduction system and more generally throughout the heart though we have not formally assessed this. Alternatively, there may be overlap in signaling, but also some differences between ric-8b and Gα_s_ and the phenotypes reflect this. However, on balance the phenotypes in the two lines are quite similar, and it is plausible that the cardiac pathology after *ric-8b* deletion is largely because of effects on Gαs and consequences for downstream signaling.

Another group has developed a similar murine line with cardiac deletion of Gαs in the adult animal (17). They performed experimental studies at later time points with 5 days of tamoxifen administration and examination of the mice 3 weeks later. They observed significant mortality with death of ∼70% of the mice before the first study point and the survivors had heart failure with pronounced cardiac remodeling including eccentric cardiac hypertrophy and fibrosis. A second study transgenically expressed a Gαs dominant negative construct in the murine heart (18). Adrenergic contractile function was impaired, but basal function was unaffected and the mice did not develop heart failure or hypertrophy. Indeed, the mice were resistant to hypertrophy.

The phenotypes bear some similarities to the phenotype resulting from the transgenic expression in the heart of a receptor coupled to inhibitory G-proteins that is responsive to a synthetic agonist (19, 20, 21). On expression, administration of the ligand and subsequent increased inhibitory G-protein signaling resulted in the mice developing cardiomyopathy and impaired contractile function (20, 21). However, the time frame for development was longer taking weeks rather than days.

### Cellular mechanisms

Our and other studies support the idea that loss of ric-8b affects Gαs signaling. The loss of contractile function is compatible with the known effects of protein kinase A on various effectors in excitation-contraction coupling including the L-type calcium channel, phospholamban, and the ryanodine receptor. However, there is also myocyte loss and increased extracellular matrix and fibrosis and how impairment of Gα_s_ signaling might account for this is less clear. To investigate this, we performed RNA-seq analysis and phosphoproteomic analysis in the cardiac ventricles after tamoxifen comparing control mice and *ric-8b (flx/flx)MCM* mice.

Despite the relatively small number of phosphopeptides that were significantly downregulated or upregulated between the two sets of mice, the analysis suggested a plausible primary mechanism. A number of myosin light chain 2 (Myl2) phosphopeptides were significantly downregulated. Myl2 is a key protein that is essential for the embryonic development of the heart (22). In the adult animal, myl2 regulates actin-myosin cross bridge cycling kinetics and calcium-dependent cardiac contraction (22, 23, 24). Myl2 activity is controlled by phosphorylation, and exercise increased phosphorylation from a basal level of 30% to 50% (25). A knockin mouse in which the phosphorylation sites were rendered inactive (S14A/S15A) had significant contractile abnormalities (26). Mutations in *MYL2* in man have been found to cause both dilated and hypertrophic obstructive cardiomyopathy (23). Thus, changes in phosphorylation and function of myl2 are a credible mechanism for the changes in contractile function observed in *ric-8b (flx/flx)MCM* mice after tamoxifen. The primary protein kinase responsible for phosphorylation of myl2 is myosin light chain kinase (23), but it is unclear how this might relate to upstream signaling mediated *via* Gαs, though it might involve PKC and∖or MAPK from the phosphoproteomic analysis.

There may also be secondary and some potentially protective cellular responses in *ric-8b (flx/flx)MCM* mice after tamoxifen. The RNA-seq data show changes in pathways underlying extracellular matrix organization consistent with the fibrosis seen in the in *ric-8b (flx/flx)MCM* mice after tamoxifen. In addition, there is support for an inflammatory response with pathways such as “leucocyte migration and chemotaxis,” “neutrophil activation,” and “cytokine and cytokine receptor” enriched. Fibrosis and inflammation are likely secondary to the myocyte damage occurring after cellular deletion of ric-8b. The phosphopeptide analysis revealed increased phosphorylation of heat shock proteins in *ric-8b (flx/flx)MCM* mice after tamoxifen, and pathway analysis identified “response to heat” and “intracellular protein transport” as being upregulated.

### Cardiac protection and drug development

In general, when considering G-protein signaling in cardiac function, the autonomic nervous system is a key focus. The vagus nerve acts to slow heart rate, and increased activity may be antiarrhythmic, enhance exercise capacity, and be cardioprotective (16, 27, 28, 29, 30, 31, 32, 33, 34). In contrast, activation of the adrenergic system while initially adaptive may later become maladaptive particularly in heart failure though the exact context may be important (12, 34, 35). In general, the actions of acetylcholine released from the vagal nerve are mediated *via* the M2 muscarinic receptor and inhibitory G-proteins (36, 37). In contrast, the actions of the sympathetic nervous system are largely mediated by the actions of noradrenaline *via* beta adrenergic receptors and actions *via* signaling entrained by the stimulatory G-protein (12). However, there are subtleties with the ability of beta2 adrenergic receptors to couple to inhibitory G-proteins and variations in receptor and G-protein expression resulting in changes in signaling (38, 39). Our results show that ric-8b and Gα_s_ signaling maintains contractile function. It is interesting that genetic deletion of beta 1 and beta 2 adrenergic receptors leads to a less severe cardiac phenotype than reported here (40) and suggests that some basal Gα_s_ signaling perhaps mediated *via* other G protein–coupled receptors is important. It is also well-known that the functioning of the sympathetic and parasympathetic nervous systems depends to a degree on an interaction between the two at both the nervous and tissue level (41, 42).

There has been interest in developing molecules that could inhibit the interaction of ric-8b with Gαs as potential new therapeutic agents (43). Superficially, it might appear that our results indicate that this could be detrimental to cardiac function. However, it is worth bearing in mind that β-blockers were initially contraindicated in heart failure. However, if introduced with careful clinical monitoring, it is now known from a number of clinical trials that they improve outcomes including a substantial effect on overall mortality (11, 44, 45). Thus, partial but not complete inhibition may be beneficial.

### Study limitations

We had to rely on genetic techniques to explore the function of ric-8b, but it would be interesting to disrupt signaling potentially using pharmacological agents. This would also allow assessment of a more graded response than is available using the cre∖loxP approaches. We also did not look longer term; studying the mice in a relatively short time window after tamoxifen administration. We used a mix of mice with a normal genotype and those carrying the cre recombinase transgene for the control population. It is probably better to use solely a cre expressing population as there have been reports of cellular toxicity particularly after tamoxifen administration (46). As detailed in the Experimental procedures, we did not see evidence for this. In addition, we have not managed to definitively assess if ric-8b has functions outside of those involving Gα_s_. Finally, despite the conservation of ric-8 proteins it would be important to confirm their role in human systems.

## Conclusion

Our studies reveal the importance of ric-8b in maintaining contractile function in cardiac myocytes. This is likely *via* actions through Gαs and phosphorylation of myl2.

## Experimental procedures

### Generation of ric-8b (flx/flx) MCM cre+ and Gα_s_ (flx/flx) MCM cre+ mice

Global deletion of *ric-8b* in the mouse is embryonically lethal (http://www.mousephenotype.org/data/genes/MGI:2682307#section-associations). A TM1c allele was generated by the International Mouse Phenotyping Consortium and Mary Lyon Centre at Medical Research Council Harwell in which exon 4 is flanked by loxP sites suitable for gene deletion in the presence of cre recombinase. The *Gα*_*s*_ *(flx* and *flx)* mice were used as previously described (47, 48) and have loxP sites placed in introns upstream and downstream of exon 1. We crossed both these lines of mice with a cre recombinase expressing murine line in which expression is driven by a tamoxifen-inducible cardiac myocyte specific cre (often referred to as mercremer (MCM)), and this was used as previously described (49, 50). Mice were maintained at Queen Mary University of London animal core facility under U.K. Home Office guidelines relating to animal welfare. All mice were kept in a pathogen-free temperature-controlled environment (21–23 °C) with 12-h day/12-h night light cycles. Animal were allowed access to standard rodent chow and water *add-libitum*. Mice were studied between 10 and 12 weeks of age. The study was approved by the Animal Welfare and Ethical Review Body at Queen Mary, University of London. Use of animals in all the studies was in accordance with the United Kingdom Animal (Scientific Procedures) Act of 1986 and procedures were undertaken under PPL 70∖7665 and PE9055EAD. Mice were ear clipped at 3 to 4 weeks of age, and genomic DNA was isolated from these. Genomic DNA was prepared, and the *ric-8b (flx, flx)* and *Gα*_*s*_ *(flx, flx)* genotyping was performed as previously described (10, 48). Identification of *Cre* was determined using the following primer sets: MCMcreF5′-ATCCGAAAAGAAAACGTTGA3′ and MCMcreR5′-ATCCAGGTTACGGATATAGT-3′. The presence of the *Cre* transgene was determined by the presence or absence of a *Cre* band (650 bp). Control animals are littermates with a mix of either a MCM positive or normal genotype in an approximately equal mix. The exact numbers are indicated in the Legends. We do not see a phenotypic difference between these two genotypes. For example, combining the echocardiography data shown in Figures 2 and 11 for these two genotypes (n = 6 for both genotypes): ejection fraction post tamoxifen: WT 59.2 ± 4.0, cre+ 61.8 ± 4.8, fractional shortening post tamoxifen: WT 32.7 ± 3.6, cre+ 34.8 ± 2.3, ejection fraction pre-tamoxifen: WT 63 ± 3.0, cre+ 65.3 ± 2.4, FS pre-tamoxifen: WT 35.2 ± 2.9, cre+ 33.2 ± 1.4. None of the comparisons are significant (all *p* > 0.9 with one way ANOVA).

### Tamoxifen injection

Tamoxifen (Sigma-Aldrich, T5648-1G) was suspended in sunflower oil (10 mg in 900 μl of sunflower oil and 100 μl ethanol) and 1 mg tamoxifen/25g body weight was injected intraperitoneally in mice for three consecutive days, and mice were studied 2 days after the last dose. Mice of both sexes were used.

### Quantitative real time-reverse transcription PCR

RNA was isolated using the RNeasy kit (Qiagen). cDNA was synthesized using the high-capacity cDNA reverse transcription kit (Thermo Fisher scientific). *ric-8b* and *Gnas* cDNA was quantified using Taqman gene expression assays (Thermo Fisher Scientific, Assay ID: Mm01224666_m1 which spans exon 3–4 for *ric-8b* and Mm03945887_s1 for *Gnas* at exon 1 boundary). In addition, quantitative real-time qPCR assay was used to assay the presence of fibrosis markers: Collagen type 1 alpha 1 (Thermo Fisher Scientific, Assay ID: Mm00801666_g1), Collagen type 3 alpha 1 (Thermo Fisher Scientific, Assay ID: Mm01254476_m1), TGF-β1 (Thermo Fisher Scientific, Assay ID: Mm01178820_m1), TGF-β2 (Thermo Fisher Scientific, Assay ID: Mm00436955_m1), and Connective tissue growth factor (Thermo Fisher Scientific, Assay ID: Mm01192933_g1). All genes were assayed in triplicate, and relative gene expression was quantified using the comparative C_T_ method with *Gapdh* as the reference housekeeping gene.

### Murine echocardiography

Echocardiography was performed using Vevo 3100 ultrasound system (VisualSonics) equipped with a scan probe head, RMV-707B, working at a frame rate of 100 frames per second and with a transducer which has a central frequency of 30 MHz. Mice were anesthetized with isoflurane (4% for induction and 1.5% for maintenance in 100% oxygen). Each animal was placed on a heating table in a supine position with the extremities fixed to the pad through surgical tape. Hair was removed from the chest, and warm ultrasound gel (Aquagel lubricating Gel from Parker Labs) applied to the thorax surface. Temperature was monitored using a rectal probe and was maintained at 37 °C ± 0.5. 2D-guided LV M-mode echocardiography is recorded from the short-axis view and/or the long-axis view at the level of papillary muscles. Measurements were made using the leading edge-to-leading edge method according to the guidelines and standards of the American Society of Echocardiography (51, 52, 53). LV wall thickness is evaluated at the interventricular septum (IVS) and the LV posterior wall (LVPW). End-diastolic measurements (interventricular septal thickness diastolic (IVSd), LV posterior wall thickness diastolic (LVPWd), and LV internal dimension diastolic (LVIDd)) are obtained at the point of maximal LV diastolic dimension. LV end-systolic dimensions (IVSs, LVPWs, and LVIDs) are obtained at the time of most anterior systolic excursion of the LVPW associated with minimal chamber dimension. All LV dimensions are presented as the average of measurements using the leading-edge technique of 3 to 5 consecutive selected sinus beats. LV systolic function is evaluated from fractional shortening (FS%), ejection fraction (EF%) and cardiac output (CO) and fractional area change (54) are then calculated from M-mode-derived LV dimensions using the formula below (55).

$$\text{EF}\%=100x\left[\left({\text{LVIDd}}^{3}-{\text{LVIDs}}^{3}\right)/{\text{LVIDd}}^{3}\right]$$

FS%=100x[(LVIDd−LVIDs)/LVIDd]

$$\text{FAC}\%=100x\left(\text{Endo}\;{\text{area}}_{d}-\text{Endo}\;{\text{area}}_{s}\right)/\text{Endo}\;{\text{area}}_{d}$$

$$\text{LV}\;\text{mass}=1.05[{\left(\text{IVSd}+\text{LVIDd}+\text{PWD}\right)}^{3}-{\text{LVIDd}}^{3}$$

### Cardiac myocyte isolation

We followed a modified version of a protocol previously described (56). Mice aged 8 to 12 weeks were heparinized and anesthetized. The chest was opened to expose the heart. Descending aorta was cut, and the heart was immediately flushed by injection of 7 ml EDTA buffer into the right ventricle. Ascending aorta was clamped using Reynolds forceps, and the heart was transferred to a 60 mm dish containing fresh EDTA buffer. Digestion was achieved by sequential injection of 10 ml EDTA buffer, 3 ml perfusion buffer, and 30 to 50 ml collagenase buffer into the left ventricle. Constituent chambers (left and right ventricle) were then separated and gently pulled into 1-mm pieces using forceps. Cellular dissociation was completed by gentle trituration, and enzyme activity was inhibited by addition of 5 ml stop buffer. Cell suspension was passed through a 70 μm filter, and then 5 ml of stop buffer was used to rinse the filter. The supernatant was put into 2 15 ml Falcon tubes and left for 20 min at room temperature to allow gravity separation. The pellets were plated onto laminin (5 μg/ml) precoated 13 mm glass coverslips, in a humidified tissue culture incubator (37 °C, 5% CO2). After 1 h, 1 ml of minimum essential medium containing 10% fetal calf serum, and 1% penicillin streptomycin was added to the cell suspension in each well of a 12-well plate containing the coverslips. EDTA buffer (in mM): NaCl 130, KCl 5, NaH_2_PO_4_ 0.5, Hepes 10, glucose 10, 2,3-butanedione monoxime 10, taurine 10, EDTA 5. Perfusion buffer (in mM): NaCl 130, KCl 5, NaH_2_PO_4_ 0.5, Hepes 10, glucose 10, 2,3-butanedione monoxime 10, taurine 10, MgCl2 1. Collagenase buffer (in mg/ml): collagenase II 0.5, collagenase IV 0.5, and protease XIV 0.05. Stop buffer was made with the perfusion buffer containing 5% sterile fetal bovine serum.

### Single-cell electrophysiology

Calcium currents were measured using the whole cell configuration of the patch-clamp technique. Patch-clamp current recordings were performed with an Axopatch 200B amplifier (Axon Instruments) using fire-polished pipettes with a resistance of 3 to 4 MΩ pulled from filamented borosilicated glass capillaries (Harvard Apparatus, 1.5 mm OD x 1.17 mm ID). Data were acquired and analyzed using a Digidata 1322A interface (Axon Instruments) and pCLAMP software (version 10, Axon Instruments, https://www.moleculardevices.com/products/axon-patch-clamp-system/acquisition-and-analysis-software/pclamp-software-suite). Membrane current data were digitized at 10 kHz, low pass filtered at 2 kHz, and were normalized to membrane capacitance. All experiments were done at room temperature.

To record calcium currents (ICa,L), we used a TEA bath solution containing (mM): TEA-Cl 140, MgCl_2_ 2, CaCl_2_ 5.4, glucose 10, Hepes 10 (pH adjusted to 7.4 with TEA-OH). The internal solution for the pipette contained (mM): CsCl_2_ 100, TEA-Cl 20, EGTA 10, Hepes 10, ATP (as magnesium salt, Sigma-Aldrich) 5, and GTP (as lithium salt, Sigma-Aldrich) 0.2 (pH adjusted to 7.3 with CsOH). Isoprenaline was diluted in water and used at a final concentration of 10 μM. Whole-cell membrane currents were elicited with 200 ms voltage steps from a holding potential of −90 mV between −80 and +80 mV in 10 mV steps.

### Histology of cardiac tissue

Mouse hearts were collected and rinsed thoroughly in PBS to remove excess blood and fixed in 10% formalin for at least 24 h. After the fixation process, they were washed twice in PBS and stored in 70% ethanol before paraffin embedding. Paraffin-embedded myocardia were cut into 5-μm-thick sections and mounted on clear Plus microscope slides. For histological analysis, sections were stained for H&E with automated Leica Autostainer XL system (Leica Biosystems) and Trichrome stain kit (Ab150686, Abcam), according to the manufacturer’s instructions.

### WGA staining

Cardiac sections were obtained as described above. After deparaffinization process, sections were rinsed thoroughly with 1× PBS and placed in dark humidified chamber to protect from light exposure. Subsequently, 50 μg/ml solution of WGA conjugated with CF-555 fluorescent dye (Cambridge Bioscience) was applied to each section with Pasteur pipette to ensure that the whole section area is covered with the solution and left in the humidified chamber at room temperature for 1 h to incubate. After incubation WGA solution was removed by gentle tilting of the slide and then washed thoroughly with double distilled water to prevent the crystallization. Sections were than left to dry in the dark for 10 min. After those sections were counterstained with 4′,6-diamidino-2-phenylindole (DAPI) for 5 min and mounted with Thermo Fisher Scientific Shandon EZ-Mount Mountant and prepared for imaging. Images were obtained using NanoZoomer S60 slide scanner (Hamamatsu) using DAPI and red filters at 20× magnification.

### TUNEL assay

Paraffin samples from mice were stained for TUNEL assay to confirm apoptosis caused by DNA fragmentation. The ApopTag Plus In Situ Apoptosis Fluorescein Detection Kit (S7111 Sigma-Aldrich) was used following the manufacturer’s instructions. The slides were kept in the dark and imaged with confocal microscopy (Zeiss LSM 880 with Airyscan Fast, Carl Zeiss). The number of TUNEL-positive cells and total number of cell nuclei counterstained with DAPI were calculated in ImageJ (https://imagej.net/ij/) with ITCN plugin (nucleus counter) to generate the % of TUNEL-positive cells (n = 3 both groups).

### Immunofluorescence of cardiac mouse tissue with ric-8b and Gα_s_ antibodies

Samples from paraffin-embedded mouse hearts were dewaxed followed by antigen retrieval with citrate buffer pH 6.0 (H-3300, Vector Laboratories) for 10 min in the microwave. Slides were then rinsed in PBS and permeabilized with 0.25% Triton in PBS for 15 min. Slides were washed several times in PBS and blocked in 5% goat serum in PBS at room temperature for 1 h. The samples were incubated overnight in PBS with 1% goat serum at 4 °C with the following antibodies: Anti-Ric-8b antibody (ab170006, Abcam) and G_α s/olf_ antibody (sc-55545, Santa Cruz Biotechnology). The slides were washed in PBS and incubated for 1 h at room temperature with 2 μg/ml of the following secondary antibodies: 488 goat anti-rabbit or 568 goat anti-mouse (A-11008 and A-11019, Invitrogen). The samples were washed with PBS and counterstained with DAPI for 5 min before the slides were mounted with a 22 mm × 50 mm coverslip with Thermo Fisher Scientific Shandon EZ-Mount Mountant (Shandon). The slides were kept in the dark and imaged with confocal microscopy (Zeiss LSM 710, Carl Zeiss). The images were processed in ImageJ.

### Staining of single ventricular myocytes with ric-8B and Gα_s_ antibodies

Following cardiac myocyte isolation protocol as described above, cells were then washed once with 1 ml of warm PBS, and 2 ml of 3% paraformaldehyde was added to each well to fix the cells and the plate was left for 20 min to incubate on a slow rocker to fix the cells onto coverslips. Cells were then washed twice with room temperature PBS for 5 min each and 2 ml of 0.2% Triton-X-100 was added to each well for 2 min to permeabilize the cells. Following this, 2 ml of blocking solution (1% w/v milk in PBS) was added to each well and the plate left to incubate for 30 min on a slow rocker. After this time the coverslips were transferred to the wet chamber (10 cm culture dish with wet filter paper inside), and cells were left to incubate overnight with the following antibodies: Anti-RIC8B antibody (ab170006, Abcam) 1 in 300 dilution and Gα s/olf Antibody (sc-55545, Santa Cruz Biotechnology) 1 in 50 dilutions. Unbound antibody was then washed off the cells with 3 x 5 min washes with PBS. In the meantime, the secondary antibody solution (1:250) was prepared by mixing 1.2 μl anti-mouse Alexa Fluor 488 secondary antibody or Alexa Fluor 594 secondary antibody (A-11008 and A-11019, Invitrogen) in 1% milk solution in 1× PBS. Fifty microliters of the mixture was added to each coverslip and wet chamber containing coverslips was put on the slow rocker for 30 min in the dark to prevent photobleaching. After that time, the cells were washed twice with 1% milk solution in PBS, once in PBS alone, and once with distilled water. The samples were washed with PBS again briefly and counterstained with DAPI for 5 min before the slides were mounted a 22 mm × 50 mm coverslip with Thermo Fisher Scientific Shandon EZ-Mount Mountant (Shandon). The slides were kept in the dark and imaged with confocal microscopy (Zeiss LSM 710, Carl Zeiss). The images were processed in ImageJ. Overlap coefficient was measured using BIOP's (Bioimaging and Optics Platform) version of JACoP (Just Another Colocalization Plugin). The plug-in will evaluate colocalization on two images according to the selected method.

### RNA sequencing

RNA from mouse ventricles, control (n = 5) and *ric-8b (flx/flx)MCM* (n = 6, ric-8b (flx/flx) combined with the tamoxifen inducible cre), 2 days after tamoxifen treatment heart RNA was isolated using the RNeasy fibrous tissue mini kit (Qiagen). RNA of a suitable concentration and purity (RIN ≥ 7) was obtained. Library preparation was carried out using NEB Next Ultra II RNA library prep kit (New England Biolabs) before sequencing on Illumina NextSeq 2000 (QMUL Genome Centre).

Resulting sequences were analyzed using Partek Flow. Contaminants were filtered and sequences quality controlled before aligning with Mm10 genome assembly using STAR (v3.7.1) and counts normalized. Differential expression of genes between *ric-8b (flx/flx)MCM* and control samples were identified using DeSeq2 (FDR < 0.05, log fold change (logFC) of ≤ −1 and ≥ 1) Significant genes of interest were deemed to have a FDR < 0.05 and a log fold change (logFC) of ≤ −1 and ≥ 1. Pathway enrichment analysis was carried out using both KEGG and Gene Ontology databases within Partek Flow. Additional KEGG pathway analysis was carried out using only upregulated or downregulated genes identified in DeSeq (FDR < 0.05, log fold change (LogFC) of ≤ −1 or ≥ 1) using DAVID (Database for Annotation, Visualization, and Integrated Discovery, www.david.ncifcrf.gov). The data have been uploaded to the gene expression omnibus repository (GSE265903).

### Phosphoproteomic analysis

Phosphoproteomics experiments were performed using MS as previously reported with some technical modifications (n = 4 for both control and *ric-8b (flx/flx)MCM*) (57, 58, 59, 60). Biopulverizer and powdered frozen tissues were lysed in 8M urea buffer supplemented with phosphatase inhibitors (10 mM Na3VO4, 100 mM β-glycerol phosphate, and 25 mM Na2H2P2O7 (Sigma-Aldrich)). Proteins were digested into peptides using trypsin as previously described (59, 60). Phosphopeptides were desalted and enriched using the AssayMAP Bravo (Agilent Technologies) platform. For desalting, protocol peptide clean-up v3.0 was used. Reverse phase-S cartridges (Agilent Technologies, 5 μl bed volume) were primed with 250 μl 99.9% acetonitrile (ACN) with 0.1% trifluoroacetic acid (TFA) and equilibrated with 250 0.1% TFA at a flow rate of 10 μl/min. The samples were loaded at 20 μl/min, followed by an internal cartridge wash with 0.1% TFA at a flow rate of 10 μl/min. Peptides were then eluted with 105 μl of 1M glycolic acid with 50% ACN and 5% TFA, and this is the same buffer for subsequent phosphopeptide enrichment. Following the Phospho Enrichment v 2.1 protocol, phosphopeptides were enriched using 5μl assay MAP TiO2 cartridges on the Assay MAP Bravo platform. The cartridges were primed with 100 μl of 5% ammonia solution with 15% ACN at a flow rate of 300 μl/min and equilibrated with 50 μl loading buffer (1M glycolic acid with 80% ACN and 5% TFA) at 10 μl/min. Samples eluted from the desalting process were loaded onto the cartridge at 3 μl/min. The cartridges were washed with 50 μl loading buffer and the phosphorylated peptides were eluted with 25 μl 5% ammonia solution with 15% ACN directly into 25 μl 10% formic acid. Phosphopeptides were lyophilized in a vacuum concentrator and stored at −80 °C (25). Phosphopeptides were desalted and enriched using the AssayMAP Bravo (Agilent Technologies) platform. For desalting, protocol peptide clean-up v3.0 was used. Reverse phase-S cartridges (Agilent Technologies, 5 μl bed volume) were primed with 250 μl 99.9% ACN with 0.1% TFA and equilibrated with 250 0.1% TFA at a flow rate of 10 μl/min. The samples were loaded at 20 μl/min, followed by an internal cartridge wash with 0.1% TFA at a flow rate of 10 μl/min. Peptides were then eluted with 105 μl of 1M glycolic acid with 50% ACN and 5% TFA, and this is the same buffer for subsequent phosphopeptide enrichment. Following the Phospho Enrichment v 2.1 protocol, phosphopeptides were enriched using 5 μll Assay MAP TiO2 cartridges on the Assay MAP Bravo platform. The cartridges were primed with 100 μll of 5% ammonia solution with 15% ACN at a flow rate of 300 μl/min and equilibrated with 50 μl loading buffer (1M glycolic acid with 80% ACN and 5% TFA) at 10 μl/min. Samples eluted from the desalting were loaded onto the cartridge at 3 μl/min. The cartridges were washed with 50 μl loading buffer and the phosphorylated peptides were eluted with 25 μl 5% ammonia solution with 15% ACN directly into 25 μl 10% formic acid. Phosphopeptides were lyophilized in a vacuum concentrator and stored at −80 °C.

Dried phosphopeptides were dissolved in 0.1% TFA and analyzed by nanoflow ultimate 3000 RSL nano instrument was coupled on-line to a Q Exactive plus mass spectrometer (Thermo Fisher Scientific). Gradient elution was from 3% to 28% solvent B in 60 min at a flow rate 250 nl/min with solvent A being used to balance the mobile phase (buffer A was 0.1% formic acid in water and B was 0.1% formic acid in acetonitrile). The spray voltage was 1.7 kV and the capillary temperature was set to 255 °C. The Q-Exactive plus was operated in data-dependent mode with one survey MS scan followed by 15 MS/MS scans. The full scans were acquired in the mass analyzer at 375- 1500 m/z with the resolution of 70,000, and the MS/MS scans were obtained with a resolution of 17,500.

MS raw files were converted into Mascot Generic Format using Mascot Distiller (version 2.8.1) and searched against the SwissProt database (SwissProt_2021_02.fasta) restricted to human entries using the Mascot search daemon (version 2.8.0). Allowed mass windows were 10 ppm and 25 mmu for parent and fragment mass to charge values, respectively. Variable modifications included in searches were oxidation of methionine, pyro-glu (N-term) and phosphorylation of serine, threonine, and tyrosine. Phosphopeptide quantification was performed using in-house software Pescal (https://www.bartscancer.london/staff/professor-pedro-cutillas/) as described before (59, 60). The resulting quantitative data were parsed into R (Version 4.2.2, https://www.r-project.org/) for further normalization and statistical analysis. The code used in analysis and visualization of data is available at https://github.com/CutillasLab/protocols2.

### Fluorescence resonance energy transfer experiments

HEK-293 cells were cultured in Modified Eagle's medium (Gibco-Invitrogen), supplemented with 100 units/ml penicillin G sodium, 100 mg/ml streptomycin (Invitrogen), and 10% fetal bovine serum. Cells were maintained in a humidified incubator at 37 °C with 95% O_2_ and 5% CO_2_. Cells were passaged up to twice a week. For FRET experiments, cells were seeded onto coverslips. Fluorescent constructs were used as previously described (10, 15). cDNA vectors were transfected using Lipofectamine 2000 (Invitrogen): Ric-8B-YFP cDNA 500 ng, Gα_s_-CFP cDNA 500 ng, Gβ_1_ cDNA 500 ng, Gγ_2_ cDNA 500 ng, β1-AR cDNA 500 ng, or CFP-YFP dimer 500 ng. The β1-adrenergic receptor in pcDNA3.1 was purchased from GenScript, ADRB1 pcDNA3.1(+)-C-eCFP.

Cells were left at 37 °C for 24 h before imaging. Then the cells were fixed onto coverslips: cells were washed once with 1 ml of warm PBS, and 2 ml of 3% paraformaldehyde was added to each well to fix the cells, and the plate was left for 20 min to incubate on a slow rocker to fix the cells onto coverslips. For experiments using β1-adrenergic receptor agonist (isoprenaline), live cells grown onto glass coverslips were incubated with isoprenaline for different periods of time (0, 5, 10, and 15 min), then put onto ice before being fixed with the same protocol as above. For FRET experiments, we used an inverted fluorescence microscope (Zeiss LSM 880 with Airyscan). To image the CFP fluorescence, we used an excitation filter of 405 nm, and an emission filter of 445 to 519 nm. For the YFP fluorescence, we used an excitation filter of 514 nm, and an emission filter of 520 to 620 nm. For the FRET channel, cells were excited with the wavelength of 405 nm and an emission filter of 520 to 620 nm. Images were captured using Plan-Apochromat 63×/1.4 Oil DIC M27 objective. Bleaching lasted for 30-min time frame, with actual bleaching starting at 10-min time frame. FRET efficiency was calculated using the following equation: FRET eff = (Dpost-Dpre)/Dpost.100 (%), D_post_ – donor intensity after the bleaching and D_pre_ – donor intensity before the bleaching. There were no bleed-through signals from CFP under YFP filter sets and *vice versa*.

### Statistical analysis

The mean and standard error of the mean are presented. The statistical tests are indicated in the legends together with the significance. Student’s *t* test (paired and unpaired) and one way ANOVA were used.

## Data availability

The murine lines are available from the International Mouse Phenotyping Consortium, Jackson Laboratories and LW. The RNA-seq datasets have been deposited at the gene expression omnibus database (GSE265903).

## Supporting information

This article contains supporting information.

## Conflict of interest

The authors declare that they have no conflicts of interest with the contents of this article.
