# Supplementary material for: The ric-8b protein (resistance to inhibitors of cholinesterase 8b) is key to preserving contractile function in the adult heart
Source: J Biol Chem. 2024 Jun 13;300(7):107470. doi: 10.1016/j.jbc.2024.107470 (PMC11277413; doi:10.1016/j.jbc.2024.107470)
Supplement: Supplementary Figure Legend [file mmc1.docx]

**Supplementary Figure.** Relative expression of fibrosis markers measured using PCR: Collagen type 1 alpha 1 (COL1A1), collagen type 3 alpha 1 (COL3A1), transforming growth factor-β1 (TGF-β1), transforming growth factor-β2 (TGF-β2) and connective tissue growth factor (CTGF) in the heart tissue of control and ric-8b (flx/flx)MCM mice. Quantitative real-time PCR in ventricular heart tissues. N=3 mice for both groups with assays performed in triplicate. * p<0.005, **** p<0.0001 one-way ANOVA, Turkey's multiple comparison test.
