# Supplementary figures and images for: The ric-8b protein (resistance to inhibitors of cholinesterase 8b) is key to preserving contractile function in the adult heart
Source: J Biol Chem. 2024 Jun 13;300(7):107470. doi: 10.1016/j.jbc.2024.107470 (PMC11277413; doi:10.1016/j.jbc.2024.107470)

## Slide 1
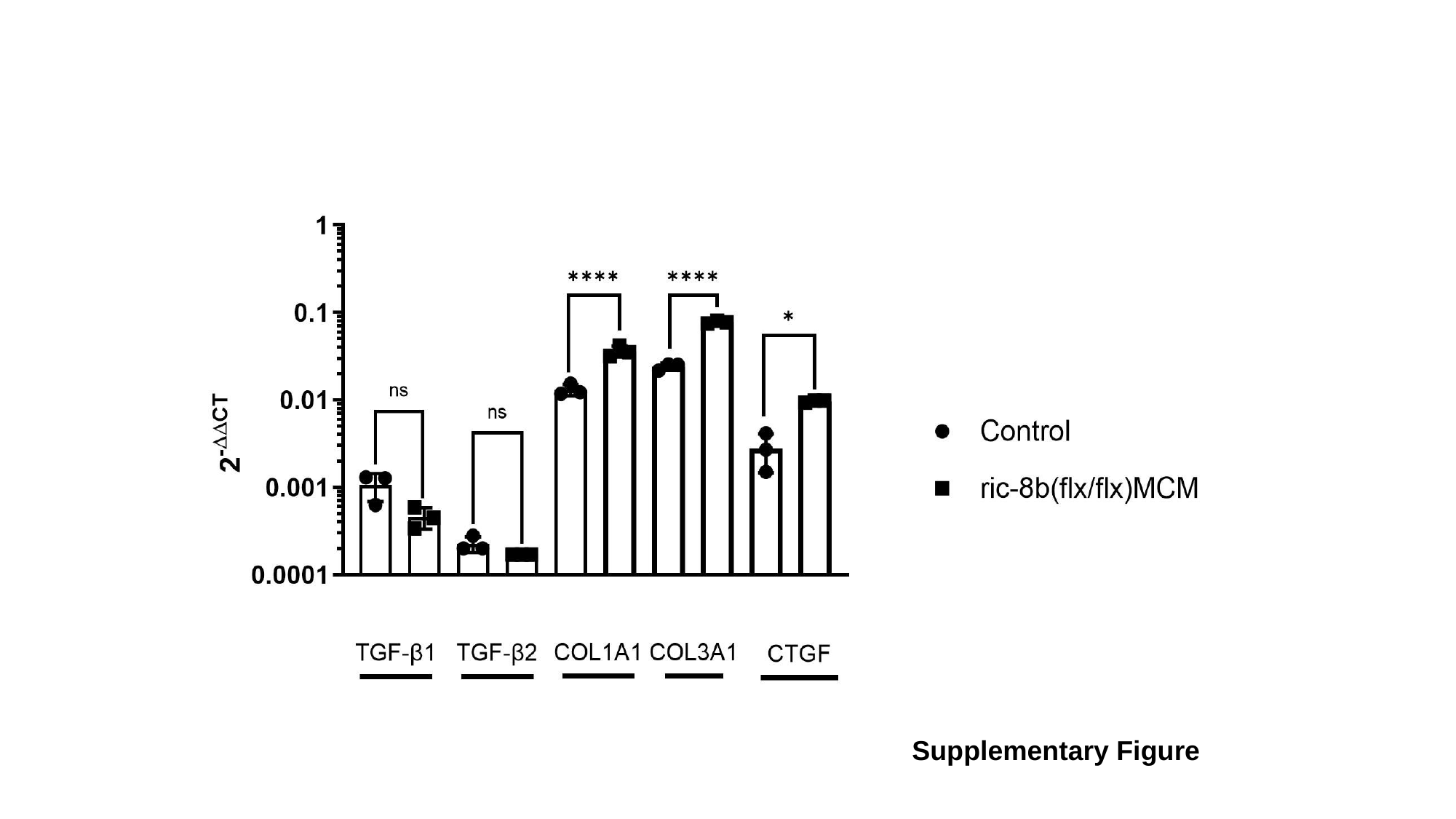

Supplementary Figure

Supplement: Supplementary Figure [file mmc2.pptx]
