# Supplementary material for: The ric-8b protein (resistance to inhibitors of cholinesterase 8b) is key to preserving contractile function in the adult heart
Source: J Biol Chem. 2024 Jun 13;300(7):107470. doi: 10.1016/j.jbc.2024.107470 (PMC11277413; doi:10.1016/j.jbc.2024.107470)
Supplement: Supplementary Tables 7 and 8 [file mmc4.docx]

| **Process** | **Normalised Enrichment** | **P-value** |
| --- | --- | --- |
| ventricular cardiac muscle tissue morphogenesis [GO:0055008] | 34.4 | <0.001 |
| heart development [GO:0007507] | 11.5 | <0.001 |
| cardiac muscle contraction [GO:0060048] | 14.1 | 0.001 |
| lipid metabolic process [GO:0006629] | 34.4 | 0.001 |
| negative regulation of reactive oxygen species metabolic process [GO2000378]. | 34.4 | 0.001 |
| regulation of the force of heart contraction [GO:0002026] | 15.9 | 0.007 |
| sarcomere organization [GO:0045214] | 9.82 | 0.017 |

**TABLE 7**

Top seven down regulated pathways (KO vs WT)

**TABLE 8**

| **Process** | **Normalised Enrichment** | **P-value** |
| --- | --- | --- |
| sarcomere organization [GO:0045214] | 15.84 | P<0.001 |
| protein kinase A signaling [GO:0010737] | 41.58 | P<0.001 |
| heart morphogenesis [GO:0003007] | 18.48 | 0.004 |
| response to heat [GO:0009408] | 16.63 | 0.006 |
| intracellular protein transport [GO:0006886] | 11.88 | 0.011 |
| cardiac muscle cell development [GO:0055013] | 11.88 | 0.012 |
| positive regulation of neuron projection development [GO:0010976] | 11.09 | 0.013 |

Top seven upregulated pathways (KO vs WT)
